# Editorial: Resource Recovery from Wastewater by Biological Technologies

**DOI:** 10.3389/fmicb.2017.00998

**Published:** 2017-06-02

**Authors:** Daniel Puyol, Damien J. Batstone

**Affiliations:** ^1^Group of Chemical and Environmental Engineering, School of Experimental Sciences and Technology, King Juan Carlos UniversityMostoles, Spain; ^2^Advanced Water Management Centre, University of QueenslandBrisbane, QLD, Australia; ^3^CRC for Water Sensitive CitiesClayton, VIC, Australia

**Keywords:** resource recovery, wastewater treatment, biological technologies, circular economy, cradle-to-cradle

Wastewater represents an opportunity to regain and re-introduce into the market valuable resources for achieving a global circular bioeconomy. Particularly in non-developed countries, cradle-to-cradle access to raw materials becomes not only an opportunity, but ultimately a human need. Considering that a substantial fraction of global nutrients, organic carbon, and metals are currently being dissipated in wastewater treatment plants, this is a present and future challenge for sustainable industrial development. This Research Topic focuses on recent advances on microbial technologies for resource recovery from urban and industrial wastewater, and on novel biotechnologies development.

This Research Topic includes a review manuscript encompassing the most attractive resources that can be extracted from wastewater (Puyol et al.). These include enabling technologies, particularly in the context of novel domestic wastewater platforms, as well as innovative microbial pathways or cooperative metabolism to obtain high value-added resources as biopolymers from organics, single-cell proteins, and third generation biofuels such as biogas, biohydrogen, or biodiesel. The recovery of metals from industrial sources (biomining) is also reviewed, particularly new methods of metals recovery from dispersed sources by biological immobilization. Finally, the impact of bioelectrochemical systems on resource recovery is analyzed from three perspectives: energy recovery by microbial fuel cells, bioproducts by microbial Electrosynthesis, and nutrients and metals recovery by specific technologies like electrodialysis.

A novel perspective for phosphorus recycle has been analyzed by Mukherjee et al. West Asia is the zone with the highest production of rice in the world. Parboiled rice mill effluent is an unnoticed source of phosphorus currently being wasted, with reported concentrations averaging 40 mg/L. A novel strategy for phosphorus recovery is proposed, where phototrophs are selected as biological drivers for phosphorus recovery via poly-phosphate accumulation. The biomass generated can be used as biofertilizers with slow-releasing nutrients properties.

Chain elongation is becoming an attractive biotechnology to produce high value-added bioproducts as medium-chain carboxylates through reversed β-oxidation metabolism. This Research Topic addresses this subject in three dedicated manuscripts. The analysis of the limitation of pH on the chain elongation has been explored by Ganigué et al. where acetogenic bacteria were used to ferment syngas from a waste gasification origin into ethanol and butanol that were subsequently chain-elongated into C6 compounds (caproate and hexanol) in the same process. While low pH seemed to trigger the production of C2 and C3 compounds, values below 4.5 strongly inhibited the reverse β-oxidation process mainly performed by *Clostridium kluyveri*. Consequently, the authors recommended to optimize the chain elongation process at a pH value of 4.5–5. The analysis of microbial dominance on complex heterogeneous mixed cultures of chain-elongating bacteria was conducted by Andersen et al. Contrarily to the current view of medium chain fatty acids (MCFA) being uniformly inhibitory to fermentation, the authors demonstrated that the varied tolerance to MCFA within the community can lead to the dominance of some species and the suppression of others, which can result in a decreased productivity of the fermentation. This was evidenced by a strong correlation between the dominance of the species *Clostridium* sp. BS-1, which can perform chain elongation to produce octanol, and the suppression of other species, as *Clostridium* sp. CPB-6, which can produce hexanoic acid, and *Lactobacillus* spp. and *Acetobacterium* sp., which generates intermediates as short chain fatty acids and light alcohols. This, in turn, causes an overall decrease of the MCFA production. A direct application of chain elongation in a real case platform has been studied by Kucek et al. In that manuscript, the authors use wine lees, which consisted primarily of settled yeast cells and ethanol from wine fermentation, as substrate to conduct continuous production of MCFA, specifically n-caprylate and n-caproate. The experimental strategy was based on direct continuous in-line extraction (through a membrane contactor) of the MCFA to increase the product recovery ratio and enhance the mass transfer. By improving the mass transfer, they achieved the highest n-caprylate-to-n-caproate product ratio reported of 1.0 (COD basis). This work also entails one of the first approach in extracting high value-added MCFA from organic waste with no external electron donor addition, and therefore implies a clear technological advance for further up-scaling.

The biodiesel industry may be a promising option to compete with crude oil if production costs can be decreased. Side products from biodiesel production are currently one of the drawbacks for the sustainability of the process. Glycerol is the main one, and can be converted into high value-added bioproducts by specialized fermentation. Roume et al. explored the use of a bioelectrochemical systems to perform the conversion of glycerol into 1,3-propanediol. As accumulation of metabolites has been an impediment for the enhancement of the process in previous BES applications, the authors propose to extract *in situ* the organic acids. This caused a positive impact on the 1,3-propanediol yield and allowed to recover propionate from glycerol fermentation, which may open up possibilities for the improvement of the economic and environmental viability of the biodiesel production.

An example where the resource can be biomass rather than waste itself is presented by Gu et al. A very specific community can be viewed as a value-added product to be used in onsite applications where the metabolic capabilities of the developed communities can be leveraged. In this work, the authors proposed to develop and enrich a phenol-degrading community that is adapted to remediate very low phenol concentrations (around 500 ug/L), and then use this enrichment immobilized on granular activated carbon as a valuable resource to be placed in drinking water biofilters, considerably reducing costs of other expensive technologies as reverse osmosis or advanced oxidation processes.

Resource recovery from wastewater is entering in a phase of technology development and upscaling. Europe is currently leading this stage as there are a wide range of technologies and startups that are being specialized on specific and highly technic processes (patent stage). It is expectable that another technological round will lead to full industrial deployment of the resource recovery from wastewater by biological technologies once the big companies on water management will enter the scenario.

## Author contributions

Both DP and DB have been participated equally in the Editorial and both are primary authors.

### Conflict of interest statement

The authors declare that the research was conducted in the absence of any commercial or financial relationships that could be construed as a potential conflict of interest.

